# Inherent Beta Cell Dysfunction Contributes to Autoimmune Susceptibility

**DOI:** 10.3390/biom11040512

**Published:** 2021-03-30

**Authors:** Yong Kyung Kim, Lori Sussel, Howard W. Davidson

**Affiliations:** 1 Barbara Davis Center for Diabetes, University of Colorado Denver Anschutz Medical Campus, Aurora, CO 80045, USA; yong.k.kim@cuanschutz.edu (Y.K.K.); lori.sussel@cuanschutz.edu (L.S.); 2 Department of Immunology & Microbiology, University of Colorado Denver Anschutz Medical Campus, Aurora, CO 80045, USA

**Keywords:** pancreatic islet, beta cell, autoimmunity, type 1 diabetes, mitochondria

## Abstract

The pancreatic beta cell is a highly specialized cell type whose primary function is to secrete insulin in response to nutrients to maintain glucose homeostasis in the body. As such, the beta cell has developed unique metabolic characteristics to achieve functionality; in healthy beta cells, the majority of glucose-derived carbons are oxidized and enter the mitochondria in the form of pyruvate. The pyruvate is subsequently metabolized to induce mitochondrial ATP and trigger the downstream insulin secretion response. Thus, in beta cells, mitochondria play a pivotal role in regulating glucose stimulated insulin secretion (GSIS). In type 2 diabetes (T2D), mitochondrial impairment has been shown to play an important role in beta cell dysfunction and loss. In type 1 diabetes (T1D), autoimmunity is the primary trigger of beta cell loss; however, there is accumulating evidence that intrinsic mitochondrial defects could contribute to beta cell susceptibility during proinflammatory conditions. Furthermore, there is speculation that dysfunctional mitochondrial responses could contribute to the formation of autoantigens. In this review, we provide an overview of mitochondrial function in the beta cells, and discuss potential mechanisms by which mitochondrial dysfunction may contribute to T1D pathogenesis.

## 1. Introduction

Type 1 diabetes (T1D) is caused by immune mediated loss of insulin producing beta cells, resulting in a life-long intrinsic inability to maintain glucose homeostasis [1]. Currently, the only treatment option is insulin replacement therapy. Although there is general acceptance of the fundamental features of the disease, many important questions still remain. In particular, while it has been known for many years that the rate of disease progression can vary considerably between individuals, precisely why this occurs remains obscure. It was originally believed that few, if any, functional beta cells remain in subjects with long-standing T1D. However, it is now evident that this assumption was false, and that in many individuals a significant number of beta cells can survive persistent autoimmunity, and produce detectable levels of insulin and C peptide decades after clinical onset [2,3]. Moreover, histological analyses of human pancreata at various stages of disease indicate that immunologic destruction does not always occur in a uniform manner [4]. These observations suggest that there may be cell-intrinsic differences between individual beta cells in their response to a proinflammatory environment, and/or susceptibility to autoimmune attack. At present this remains largely a matter of conjecture, although there is clear evidence that abnormal beta cells accumulate during prediabetes in both animal models [5,6] and in humans [7]. However, whether they mainly represent a pathological change that enhances autoimmunity [8] or reflect a protective response to reduce “collateral damage” during islet inflammation, remains uncertain. 

**Figure 1:**
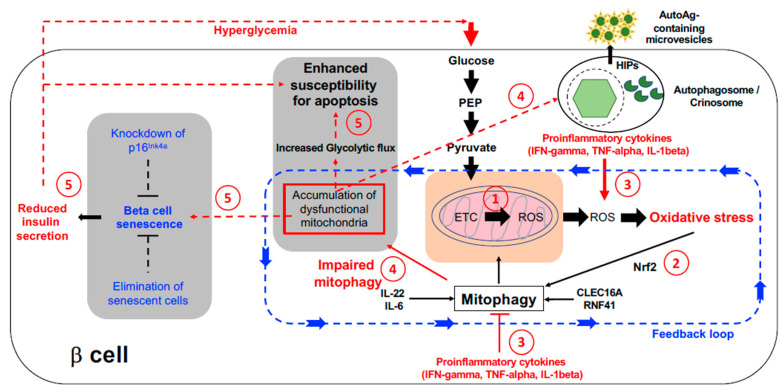
Possible contribution of mitochondrial dysfunction to beta cell failure in T1D. (**1**) Oxidative phosphorylation leads to Reactive Oxygen Species (ROS) production that can cause oxidative stress and lead to damage. (**2**) Oxidative stress triggers NRF activation and protective responses including an increase in mitophagy. (**3**) Exposure to proinflammatory cytokines or other pathogenic stressors will increase the level of ROS exposure that may then overload the detoxification machinery. (**4**) Impaired mitophagy will lead to accumulation of dysfunctional mitochondria and a global inhibition of flux through endo-lysosomal pathways which may promote the generation and/or secretion of immunogenic microvesicles containing autoantigens such as Hybrid Insulin Peptides (HIPs), forming a toxic positive feedback loop. (**5**) Over time the adaptive mechanisms to promote beta cell survival will become irreversible, resulting in beta cell dysfunction, increased senescence, reduced insulin secretion, and ultimately apoptosis. Solid lines indicate known pathways; dotted lines indicate putative pathways. Additional abbreviations: Phosphoenolpyruvate (PEP); Electron Transport Chain (ETC)).

Mitochondria play a central role in beta cell homeostasis, and there is abundant evidence that their dysfunction is critical to the development of type 2 diabetes (T2D) [9]. Mitochondrial dysfunction has also been implicated in T1D pathogenesis, mainly in the context of altered immune cell activity [10] and generation of reactive oxygen species (ROS) that could induce and/or potentiate oxidative stress [11]. Mitochondrial ROS is a necessary by-product of the central function of beta cells to secreted insulin in response to glucose. Beta cells possess multiple mechanisms to mitigate the toxic effects of ROS that are controlled by the transcription factor NF-E2-Related Factor 2 (NRF2; also known as Nuclear factor, erythroid 2 like 2 (NFE2L2) [12]; however, mitochondrial ROS may still represent a potential source of vulnerability to cause exacerbation of pathological conditions. In this review we summarize the central role that mitochondria play in beta cells, and discuss potential mechanisms by which mitochondrial function and dysfunction in endocrine and immune cells may contribute to T1D pathogenesis (Figure 1).

## 2. Mitochondrial Are Critical to Glucose Stimulated Insulin Secretion

The primary function of beta cells is to release insulin, for example in response to elevated blood glucose levels after a meal. Mitochondria play a critical role in this process. First, mitochondrial ATP synthesis from oxidative metabolism of glucose or other nutrients provides the energy to power membrane depolarization and granule exocytosis. Second, other mitochondrial metabolites such as glutamate, citrate, NAD(P)H, and GTP, are required to amplify this signal to provide maximal stimulated insulin secretion (GSIS) [13]. Glucose enters beta cells by facilitated transport mediated by GLUT1 & GLUT3 (*SLC2A1*/*SLC2A3*) in humans, or GLUT2 (*Slc2A2*) in rodents, and is then converted to pyruvate by glycolysis [14,15]. Pyruvate then enters mitochondria and is either decarboxylated by pyruvate dehydrogenase (PDH) to generate acetyl-CoA, or carboxylated by pyruvate carboxylase (PC) to generate oxaloacetate (OAA) [16]. Acetyl-CoA is consumed by the tricarboxylic acid (TCA) cycle that together with the coupled electron transport chain (ETC) generates ATP by oxidative phosphorylation (OxPhos) [17]. In contrast OAA is converted to phosphoenolpyruvate (PEP) by mitochondrial PEP carboxykinase and is then exported back to the cytoplasm. The increase in the cytosolic ATP/ADP ratio following glucose metabolism leads to closure of ATP-dependent K^+^-channels (K_ATP_), triggering membrane depolarization, activation of voltage-gated Ca^2+^-channels [18], and Ca^2+^-dependent exocytosis of the contents of insulin secretory granules [19]. It has generally been thought that OxPhos is the primary source of ATP driving K_ATP_ closure. However, this has recently been challenged by Lewandowski and colleagues who concluded that the ATP is mainly generated by local metabolism of PEP by plasma membrane associated pyruvate kinase (PK) that has been recruited adjacent to the channels by the allosteric activator fructose bisphosphate [20]. In a revised model they propose that during GSIS, mitochondria oscillate between anaplerotic and oxidative states regulated by the availability of ADP for OxPhos. Initially ADP is depleted by PK, activating the mitochondrial PEP cycle, and providing additional substrate for ATP synthesis by PK. This “triggering” phase continues until the increased hydrolysis of ATP by ion pumps and components of the exocytotic machinery has generated sufficient ADP, whereupon OxPhos resumes, providing the energy required to sustain membrane depolarization and power exocytosis during the “secretory” phase of the cycle. Thus insulin secretion is tightly coupled both to glycolytic flux and beta cell mitochondrial function [9].

## 3. Mitochondrial ROS—An Achilles Heel?

Both T1D and T2D are characterized by a loss of beta cell function, and enhanced beta cell death is a key event in the pathogenesis of both diseases. Beta cells are considered to be particularly sensitive to the induction of oxidative stress due to their low expression of detoxifying enzymes such as catalase and glutathione peroxidase [21]. Instead, they rely mainly on an alternative antioxidant system based on peroxiredoxins and thioredoxin [22]. This system has sufficient capacity to rapidly detoxify the levels of reactive oxygen species (ROS) that are generated as a by-product of mitochondrial OxPhos under physiological conditions. However, by placing a significant basal load on the detoxification machinery, mitochondrial ROS (mtROS) may also represent an “Achilles heel” for the beta cell under pathological induction of oxidative stress [23]. For example, mitochondrial hyperactivity due to persistent hyperglycemia will lead to elevated ROS production which might then exceed the detoxifying capacity of the cell, leading to dysfunction, and ultimately cell death [11]. Similarly, high mtROS may also make beta cells particularly sensitive to ROS generated in response to a proinflammatory environment. Unsurprisingly, mitochondria are both a major source of ROS and also a primary target of ROS attack [23], which may significantly impair their function. Consequently, it is critical to beta cell function and survival that elevated ROS also leads to rapid induction of multiple cytoprotective antioxidant pathways. This is achieved by disruption of KEAP1-containing complexes, which allows nuclear translocation of the key transcription factor NRF2 [12]. Numerous studies illustrate the critical role that NRF2 activation plays in beta cell biology. For example, islets from *Nrf2^-/-^* mice exposed to oxidative stress in vitro have lower levels of antioxidant enzymes and decreased survival compared to wild-type islets [24]. Conversely, treatment of human islets with NRF2 activators protected them from apoptosis in response to prolonged oxidative stress [25].

## 4. Regulation of Mitochondrial Autophagy (Mitophagy) in Beta Cells

Autophagy induction is a key mechanism of NRF2-dependent cytoprotection [12]. It is one of the major mechanisms for recycling cellular components in eukaryotes, providing both a source of intracellular nutrients and a method to remove protein aggregates, and damaged or unwanted organelles. This is achieved by targeting the unwanted components to endo-lysosomes, where they are then degraded [26]. Autophagy is a global term for 3 mechanistically distinct pathways, namely macroautophagy, microautophagy, and chaperone-mediated autophagy [27,28,29]. As their names suggest, each target components of distinct sizes. Thus, macroautophagy primarily targets intact organelles such as mitochondria and insulin secretory granules, whereas microautophagy recycles much smaller complexes such as cytoplasmic protein aggregates, and chaperone-mediated autophagy targets a select subset of soluble cytoplasmic proteins [30]. Although originally considered as largely nonselective, it is now clear that the targeting of organelles for autophagy is tightly regulated [31]. This has led to sub-classifications such as mitophagy [32,33] and ER-phagy [34] to refer to autophagy of a specific organelle class. Defunct insulin secretory granules can also be recycled by the related process of crinophagy [35,36] that involves their direct fusion with lysosomes. 

Mitochondria are highly susceptible to damage from ROS and other forms of cellular stress [37] that can lead to dysfunction and subsequent loss of organelle integrity. Since this may ultimately trigger apoptosis [38,39], mitophagy provides a critical protective mechanism, and is strongly induced by oxidative stress. Due to this important function, there are several lines of evidence linking mitophagy to inflammation and the autoimmunity underlying the pathogenesis of several autoimmune diseases, including T1D [40,41]. Mitophagy is initiated by the sensor kinase PINK1 [33], which accumulates on the outer membrane of damaged mitochondria due to impaired import of newly synthesized molecules. Reduced import allows dimerization and auto-phosphorylation to form the fully active enzyme. Active PINK1 then phosphorylates serine-65 in the ubiquitin-like domain of the E3 ubiquitin ligase PARKIN, and an analogous residue in free ubiquitin, converting PARKIN to the active conformation and enabling tethering to mitochondria via phospho-ubiquitin modified surface proteins [42,43,44]. Active PARKIN then polyubiquitinates multiple mitochondrial outer membrane proteins, creating the substrates for autophagy receptors such as NDP52 and OPTN that promote phagophore formation around the damaged organelle [33]. The closed autophagosome then fuses with lysosomes to complete the process [26,31]. In addition to mitophagy, defective mitochondria can also be salvaged by selective removal of damaged proteins/protein complexes in mitochondria-derived vesicles (MDVs) that bud from the outer membrane [33]. PINK1 and PARKIN are also required to generate MDVs, but their subsequent delivery to late endosomes/lysosomes for degradation uses a distinct mechanism to mitophagy that is independent of the core autophagic machinery. Excess mitophagy is also detrimental to beta cells, and so recruitment of autophagy receptors to mitochondria is also regulated by deubiquitinases such USP15, USP30, and USP35 that can reverse PARKIN-mediated chain extension [33]. 

## 5. Association between Decreased Mitophagy and Diabetes

Given its central role in triggering mitophagy it is unsurprising that PARKIN is regulated at multiple levels. Besides PINK1 mediated phosphorylation, in beta cells a tripartite complex involving CLEC16A, RNF41 (also known as NRDP1), and USP8 also plays a critical role [45]. This they achieve by regulating PARKIN stability and auto-inhibition by balancing the nature and extent of its ubiquitination. Thus, RNF41 is an E3 ubiquitin ligase that can catalyze K48-linked polyubiquitination, targeting its substrates, including PARKIN, for proteasomal degradation [46,47,48]. In contrast, USP8 is a deubiquitinase that removes auto-inhibitory K6-linked ubiquitin residues from PARKIN, promoting translocation of the active enzyme to the mitochondrial membrane [33]. Perhaps paradoxically, USP8 also stabilizes active RNF41 by removing self-introduced K48-linked ubiquitin chains that would otherwise target it for degradation [49]. Furthermore, the RNF41/USP8 complex is itself stabilized by the action of the third member of the complex, CLEC16A, which is another E3 ligase and catalyzes the addition of non-degradative ubiquitin chains to RNF41 [45]. CLEC16A also promotes the accumulation of inhibitory K6 chains on PARKIN, suggesting that it might also directly influence USP8’s substrate specificity. Thus, the combined actions of the tripartite complex provide a post-translational mechanism to control cellular PARKIN levels under “healthy” conditions, and its modulation a potential method to rapidly increase mitophagy in response to mitochondrial distress [45]. However, disruption of this key regulatory network can have potentially catastrophic results on cell function. This is perhaps best illustrated by mice with a beta cell specific knockout of *Clec16A*. Islets from these animals show hyper-expression of PARKIN, reduced glucose stimulated insulin secretion, and mitochondria that are structurally and functionally abnormal [50]. These defects are accompanied by the accumulation of aberrant autophagic vacuoles containing partially degraded organelles, suggesting that the final steps of mitophagy are inhibited in the beta cells of these animals. The block in turnover can be rescued by overexpression of RNF41, indicating either that RNF41 has a direct role at both early and late stages of mitophagy, or that CLEC16A (which is localized to endosomal membranes) has a major role in regulating autophagic flux, and that its loss reduces the efficiency of macroautophagy allowing the accumulation of damaged organelles. It should also be noted that due to the loss of the PARKIN target mitofusin2 (MFN2), which also plays a critical role in ensuring the correct temporal sequence of the ER stress response, prolonged disruption of mitophagy will also exacerbate ER stress and lead ultimately to increased cell death [51]. These observations have a direct relevance to human disease since there is accumulating evidence that polymorphic variants of PINK1, PARKIN, CLEC16A and PDX1 (which regulates CLEC16A expression) show clear associations with the risk of developing autoimmune diseases such as T1D, multiple sclerosis and systemic lupus erythematosus (SLE) [41,50,52,53,54]. In general the risk SNPs have only a modest effect on expression and/or function, but there is compelling evidence that the common T1D associated SNP rs12708716 in the CLEC16A gene influences its expression in a dose dependent fashion, and that the significantly reduced expression in subjects homozygous for the risk allele correlates with impaired beta cell function [50]. Thus, the feedback loop induced by oxidative stress that links mitochondrial dysfunction and autophagy is critical for both beta cell function and survival, and a potential therapeutic target for both T1D and T2D.

## 6. Immune-Mediated Beta Cell Destruction and Autophagy

Proinflammatory cytokines, such as tumor necrosis factor-alpha (TNF-α), interleukin-1 beta (IL-1β) and interferon-gamma (IFN-γ) are implicated in T1D pathogenesis as triggers of ROS production and pathogenic ER stress in rodent models [55,56]. Although similar processes are known to exist in human disease, less is known about the involvement of specific cytokines. Furthermore, the precise role of autophagy in this process remains controversial. In a rat model, islets exposed to IFN-γ and IL-1β activated AMPK in response to ER stress, leading to reduced autophagic flux and impaired lysosomal function that contributed to beta cell apoptosis [57,58]. These findings were supported in a more recent study that provided evidence that autophagy is impaired in the islets of both humans and mice with T1D [59]. This study also showed that there was an accumulation of defective lysosomes in the beta cells of autoantibody positive donors. Conversely, interleukin-22 (IL-22) a member of the IL-10 cytokine family and interleukin-6 (IL-6) a pleiotropic pro-inflammatory cytokine were shown to stimulate autophagy to promote beta cell survival [60,61]. Thus, it is likely that cytokines can play both positive and negative roles in regulating autophagy and beta cell destruction, depending on the timing and degree of alterations in autophagic flux and the metabolic context in which the different molecular pathways become activated. This may in part be regulated by the ability of beta cells to produce nitric oxide, which both reversibly inhibits beta cell secretory function and caspase activation, and activates multiple pro-survival responses (reviewed in [62]). It is only after beta cells no longer produce nitric oxide, which occurs following prolonged exposures to cytokines of 36 h or longer [63], that the toxic actions of cytokines become irreversible and beta cells are committed to death [63,64].

## 7. Mitochondria and Senescence in the Beta Cell

Cellular senescence is a complex regulated response to stress that is characterized by proliferative arrest, and is often activated in damaged or aging cells [65,66]. In addition to cell cycle inhibition, senescent cells undergo metabolic and epigenetic changes leading to hypertrophy, increased lysosomal content, a senescence-associated secretory profile (SASP), and dysfunctional mitochondria that produce high levels of ROS [66]. Although persistent senescence can negatively impact tissue growth and cause a proinflammatory response, transient senescence programs are important in embryonic development, and can help eliminate damaged cells to improve overall tissue survival and function. This duality of roles is evident in beta cells, where cell-cycle arrest can promote both post-natal beta cell maturation and age-related loss of function following prolonged insulin resistance and pathogenic stress. In both mouse and human islets, aging is associated with an increased frequency of beta cells expressing markers of senescence such as p16^Ink4a^ and elevated beta-galactosidase activity. This is exacerbated in subjects with T2D, suggesting that increased senescence is linked to disease pathophysiology. Consistent with this hypothesis, induction of senolysis (removal of senescent cells) in mice with drug or diet induced insulin resistance improved glucose metabolism and beta cell function [67]. Conversely, ectopic expression of p16^Ink4a^ in beta cells enhances glucose-stimulated insulin secretion, likely due to mTOR activation triggering increased mitochondrial function and glucose uptake, and potentiates beta cell maturation [68]. Furthermore, elimination of p16^Ink4a^ from beta cells increases beta cell mass but negatively affects both insulin secretion and mitochondrial function [68]. Together this suggests that during aging the beneficial effect of p16^Ink4a^ -induced cellular changes on GSIS is balanced by the limitation it imposes on the ability of beta cells to undergo adaptive replication [69], and that prolonged exposure to the SASP and/or other sources of cell stress will eventually lead to exhaustion and ultimately cell death [66]. The adverse effects of senescence may also play a role in the pathogenesis of T1D. Thus, Thompson and colleagues reported that treatment of NOD mice with senolytic drugs protected them from spontaneous disease [70]. The precise mechanisms remain uncertain, but likely stem from the pro-inflammatory nature of the SASP.

## 8. Contribution of Dysregulated Autophagy to Epitope Spreading

T1D results from a loss of immune tolerance to beta cells, with epitope spreading, evidenced by the appearance of multiple autoantibodies, being a key step in pathogenesis (reviewed in [71]). How tolerance is lost is still an open question, but there is increasing evidence that neo-antigens, whose generation may be induced or increased by beta cell responses to cellular stress, likely play a key role in disease progression [56,72,73]. One example are hybrid insulin peptides (HIPs) that are attracting considerable attention as pathogenic targets of CD4+ T cells [74]. These peptides are not genetically encoded, but instead are derived by fusion of two distinct protein fragments via transpeptidation [75], and generated mainly in beta cell autophagosomes and/or crinophagic bodies during turnover of damaged or defunct secretory granules [75,76,77]. In healthy cells newly formed HIPs will likely be relatively short-lived due to the actions of lysosomal amino- and/or carboxypeptidases also present in the same compartments. However, the pathogenic changes in gene expression, autophagic flux and lysosomal function that result from cytokine stress and beta cell senescence [59,66,78] may conspire to make HIPs, and other post-translationally modified granule peptides, much more accessible to the immune system. For example, there is an increasing awareness that multiple classes of secreted microvesicles play important roles in cell–cell communication in both health and disease (reviewed by [79,80]). Many of these are derived from elements of the endo-lysosomal pathway, including autophagosomes [81] and can contain autoantigens [82] including HIPs [77] that could be captured by immune cells to promote either tolerogenic or immunogenic responses [83]. Moreover, insulitis is associated with aberrant expression of MHC class II molecules by a subset of beta cells [78]. Peptide loading occurs in endo-lysosomal compartments (including autophagosomes) [84], thus in some cytokine-stressed beta cells, HIP-MHC class II complexes may be formed, both protecting these peptides from degradation [85], and allowing direct [86] or indirect [87] presentation to pathogenic T cells.

## 9. Summary

Although T1D is an autoimmune disease that is driven by a dysfunctional immune response, Genome Wide Association Studies (GWAS) have identified a number of T1D susceptibility alleles that map to genes expressed in beta cells, prompting speculation that the beta cells themselves contribute to T1D susceptibility and/or pathogenesis [88]. In this review, we have attempted to describe the unique metabolic features of the beta cell that allows it to efficiently secrete insulin in response to glucose stimulation and rapidly adapt to changing nutritional conditions. Of these beta cell-specific processes, specialized mitochondrial functions are especially instrumental in optimizing the performance of the beta cell; however they also create an environment that is poised for metabolic dysfunction and adaptive responses often become maladaptive over time. These studies support the premise that beta cells participate in and/or propagate their destruction in the face of an autoimmune assault. Further studies will be necessary to determine the extent to which mitochondrial dysfunction also plays a role in the formation of autoantigens to promote an autoimmune response. 

## Data Availability

Not applicable.
